# Sleep complaints in individuals with subjective cognitive decline from a south‐brazilian cohort

**DOI:** 10.1002/alz70857_106854

**Published:** 2025-12-25

**Authors:** Gabriela Raquel Paz Rivas, Barbara Loeblein Uebel, Victória Tizeli Souza, Simone Sieben da Mota, Bruno De Oliveira De Marchi, Guilherme Da Silva Carvalho, Haniel Bispo De Souza, Lucas Bastos Beltrami, Rhaná Carolina Santos, Sarah Vitoria Bristot Carnevalli, Ana Letícia Amorim de Albuquerque, Leonardo Martins de Paula, Manuella Edler Zandoná Giordani, Wyllians Vendramini Borelli, Giovanna Carello‐Collar, Marcia L. Chaves, Eduardo R. Zimmer, Raphael Machado Castilhos

**Affiliations:** ^1^ Universidade Federal do Rio Grande do Sul, Porto Alegre, RS, Brazil; ^2^ Hospital de Clínicas de Porto Alegre, Porto Alegre, RS, Brazil; ^3^ Hospital de Clínicas de Porto Alegre, Porto Alegre, Rio Grande do Sul, Brazil; ^4^ Federal University of Rio Grande do Sul, Porto Alegre, Rio Grande do Sul, Brazil; ^5^ Universidade do Vale do Rio dos Sinos, São Leopoldo, Rio Grande do Sul, Brazil; ^6^ Universidade Federal de Santa Catarina, Florianópolis, Santa Catarina, Brazil; ^7^ Universidade Federal do Rio Grande do Sul, Porto Alegre, Brazil; ^8^ Clinical Hospital of Porto Alegre, Porto Alegre, Rio Grande do Sul, Brazil; ^9^ Hospital Moinhos de Vento, Porto Alegre, Rio Grande do Sul, Brazil; ^10^ Brain Institute of Rio Grande do Sul (InsCer), PUCRS, Porto Alegre, Rio Grande do Sul, Brazil; ^11^ Hospital de Clinicas de Porto Alegre, Porto Alegre, RS, Brazil; ^12^ UFRGS, Porto Alegre, Brazil; ^13^ McGill University, Montreal, QC, Canada; ^14^ Brain Institute of Rio Grande do Sul ‐ Pontifícia Universidade Católica do Rio Grande do Sul, Porto Alegre, Rio Grande do Sul, Brazil; ^15^ Universidade Federal do Rio Grande do Sul, Porto Alegre, Rio Grande do Sul, Brazil

## Abstract

**Background:**

Subjective cognitive decline (SCD) is defined as self‐perceived cognitive impairment in individuals who remain cognitively unimpaired (CU). SCD is considered an early manifestation within the continuum of neurodegenerative diseases, its etiology is heterogeneous and not fully elucidated. Emerging evidence suggests that sleep disturbances may contribute to cognitive complaints and objective cognitive decline. This study aims to investigate sleep‐related complaints in individuals with SCD evaluated from a South‐Brazilian BRASCODE cohort.

**Methods:**

We included CU individuals aged ≥65 years‐old from the BRASCODE cohort who reported subjective cognitive complaints. The exclusion criteria were: presence of uncontrolled psychiatric or medical conditions, diagnosis of cerebrovascular disease, and use of psychotropic medications. Participants underwent neurocognitive and neuropsychiatric evaluation, consisting of Memory Complaint Scale (MCS), Subjective Cognitive Decline Scale (SCD‐S), Mini‐Mental State Examination (MMSE), Geriatric Anxiety Inventory (GAI), and Geriatric Depression Scale (GDS). Sleep quality and behavioral symptoms were assessed using the Pittsburgh Sleep Quality Index (PSQI), Mild Behavioral Impairment (MBI) checklist, and Neuropsychiatric Inventory (NPI). This analysis includes participants who completed the 12‐month follow‐up. Correlations between continuous variables were assessed, with *p* <0.05 considered significant.

**Results:**

A total of 100 participants enrolled between March, 2022 and November, 2023 completed the 12‐month evaluation. Among these, 75% were women (*n* = 75), with a median age of 71 years (68–74,25) and a median of 15 years of formal education (11–18). Median scores and interquartile ranges (IQR) for the assessed scales were as follows: SCD‐patient scale = 7 (6–9); SCD‐informant scale = 5 (3–7); PSQI = 6 (4–9); GAI = 5 (2–8,75); GDS = 2 (1–4); MMSE = 29 (27–30); NPI = 1 (0–4); MBI‐patient = 3 (0–7,25); and MBI‐informant = 3 (1–7,75). A significant positive correlation was observed between PSQI and GAI scores (*p* = 0.01, rho = 0.25). No significant associations were found between PSQI and the SCD‐patient or SCD‐informant scales, MMSE scores, age, education level, or behavioral scales (MBI and NPI).

**Conclusion:**

The correlation between PSQI and GAI scores suggests that poor sleep quality in CU individuals with SCD from a South‐Brazilian cohort is linked to anxiety. Further studies may clarify the relationship between cognition, anxiety, and sleep disturbances.

